# Instruments for the assessment of patient adherence to oral anticoagulation with warfarin protocol for a systematic review

**DOI:** 10.1097/MD.0000000000017323

**Published:** 2019-10-18

**Authors:** Marcus Fernando da Silva Praxedes, Mayara Sousa Vianna, Waleska Jaclyn Freitas Nunes de Sousa, Frederico Bartolazzi, Vânia Eloisa de Araújo, Maria Auxiliadora Parreiras Martins

**Affiliations:** a Centro de Ciências da Saúde, Universidade Federal do Recôncavo da Bahia, Santo Antônio de Jesus, Bahia; b Faculdade de Farmácia, Universidade Federal de Minas Gerais, Belo Horizonte; c Faculdade de Medicina and Hospital das Clínicas, Universidade Federal de Minas Gerais, Belo Horizonte, Minas Gerais, Brazil.

**Keywords:** anticoagulants, medication adherence, validation studies, warfarin

## Abstract

**Background:**

Non-adherence can be highlighted as one of the main contributors to the occurrence of adverse events in patients treated with warfarin. The usefulness of self-reporting measures of drug adherence could be improved by following psychometric properties in the development of the measurement scales. Thus, we aimed to describe the protocol of a systematic literature review designed to investigate and describe validated instruments used to assess adherence to warfarin therapy.

**Methods:**

We will perform a systematic review will include observational and experimental studies involving the use of validated instruments to assess adherence to warfarin therapy. Dimensions of adherence raised by the selected studies will be extracted to be compared. We will systematically search electronic databases including MEDLINE, LILACS, EMBASE, and Cochrane Library using a comprehensive strategy from inception to June 31, 2019. Two reviewers will revise the literature independently using a standardized form and assess the potential bias. After the comparison of results, discrepancies will be solved after the analysis of a third reviewer.

**Result:**

The development of the present systematic will help to summarize and evaluate the validated instruments that have been previously published to assess adherence to warfarin therapy.

**Conclusion:**

This review will substantiate the discussion of relevant topics that should be assessed while providing care to patients taking warfarin. This knowledge will enable a comprehensive approach for healthcare professionals to improve treatment outcomes and the design of future investigations.

**Registration:**

The systematic review is registered in the PROSPERO international prospective register of systematic review (PROSPERO# CRD42019128324).

## 1 Introduction

Warfarin is an oral anticoagulant extensively used worldwide, especially in low- and middle-income countries.^[1]^ This coumarinic derivative is indicated for primary and secondary thromboprophylaxis. Although recognized as an important therapeutic alternative, warfarin presents as disadvantages a narrow therapeutic index and increased risk for adverse reactions, mainly represented by bleeding. Oral anticoagulation control is challenging due to the wide variability in dose-response that contributes to the unpredictability of drug effect.^[2,3]^

Therefore, strategies to control oral anticoagulation should consider aspects that may interfere with treatment success. Among these aspects, physiological factors, interactions with other medications, variations in the intake of food containing vitamin K, patient knowledge on drug therapy, genetic characteristics and treatment adherence should be taken into account while providing patient care.^[1,4,5]^

Non-adherence can be highlighted as one of the main contributors to the occurrence of adverse events, such as thromboembolism and bleeding in warfarin users. A prospective cohort study was developed in the United States involving 22,425 adults with atrial fibrillation and venous thromboembolism to investigate adherence warfarin therapy. The authors found non-adherence in 21% of studied patients which has been related to the level of education (odds ratio (OR) 1.8 (95%CI 1.2–2.7)), mental health condition (OR 1.4 (1.1–1.6) and impaired cognition (OR 2.9 (1.7–4.8)).^[6]^

According to the World Health Organization, drug adherence is the degree to which the behavior of a person represented by medication intake, diet follow-up, lifestyle changes corresponds and agrees with the recommendations of a physician or other health professional.^[7,8]^ Self-reporting has been referred as a valid method to assess adherence behaviors, despite its tendency to overestimations. They are widely used in research and clinical practice, given that clinical interview is the most accessible way for health professionals. In addition, self-report measures may help evaluating the reasons and levels of non-adherence.^[9,10]^ Although several instruments to measure self-reported adherence are available, few were subjected to the assessment of convergent validity or criteria, internal consistency and reliability.^[11]^ The usefulness of self-reporting measures of drug adherence could be improved by following psychometric properties in the development of the measurement scales.

The assessment of adherence to warfarin therapy should consider the specificities of this type of treatment that requires from patients beyond adherence to dosing regimen, frequent international normalized ratio (INR) monitoring, knowledge on dietary vitamin K intake and recognition of signs of adverse events. In addition, there is a scarcity of validated instruments adapted to this broad approach, assessing patient behavior towards warfarin therapy. Thus, we aimed to describe the protocol of a systematic literature review designed to investigate and describe validated instruments used to assess adherence to warfarin therapy.

## 2 Methods

### 1.1 Study registration

This systematic review protocol has been registered on PROSPERO as CRD42019128324. Available at: https://www.crd.york.ac.uk/prospero/display_record.php?RecordID=128324

This protocol was developed in accordance with the Preferred Reporting Items for Systematic Reviews and Meta-analysis Protocol (PRISMA).^[12]^ No ethic approval is needed for the development of this study because it will not involve individual patient data.

### 1.2 Eligibility criteria

#### 1.2.1 Types of studies

This study will include observational and experimental studies involving the use of validated instruments to assess adherence to warfarin therapy.

Inclusion criteria

- Studies involving validated instruments used to assess adherence to warfarin therapy;
- Patients aged 18 years or older;
- Patients taking warfarin for more than 3 months;
- Any indication for chronic oral anticoagulation.

Exclusion criteria

- Duplicate articles;
- Narrative or systematic review and meta-analysis;
- Case reports/series;
- Experimental studies involving animals.

#### 1.2.2 Types of interventions

*Intervention(s), exposure(s):* Studies involving validated instruments used to assess adherence to warfarin therapy.

*Comparator(s)/control:* This review will involve the selection of articles including validated instruments used to assess adherence to warfarin therapy. Dimensions of adherence raised by the selected studies will be extracted to be compared.

#### 1.2.3 Type of outcomes

The primary outcome will be the assessment of validated instruments used to assess adherence to warfarin therapy. The secondary outcomes involve the identification of the level of adherence to warfarin treatment, risk factors and consequences related to non-adherence.

### 1.3 Strategy of literature searches

We will systematically search electronic databases from inception to June 31, 2019, including MEDLINE, LILACS, EMBASE, and Cochrane Library. The research strategy will combine indexing terms and text words. There will be no date and language restriction. Additional references can be added after examination of the list of references cited by the selected articles. Search strategy of PubMed was as follows: ((’questionnaire’/exp OR questionnaire OR ’validation study’/exp OR ’validation study’ OR 'surveys and questionnaires’/exp OR 'surveys and questionnaires’) AND (’patient compliance’/exp OR ’patient compliance’ OR ’treatment adherence and compliance’/exp OR ’treatment adherence and compliance’ OR ’medication adherence’/exp OR ’medication adherence’) AND (’warfarin’/exp OR warfarin OR ’anticoagulant agent’/exp OR ’anticoagulant agent’)). This strategy served as a basis for the electronic search in the other databases.

### 1.4 Data collection and analysis

#### 1.4.1 Selection of studies

**Figure 1:**
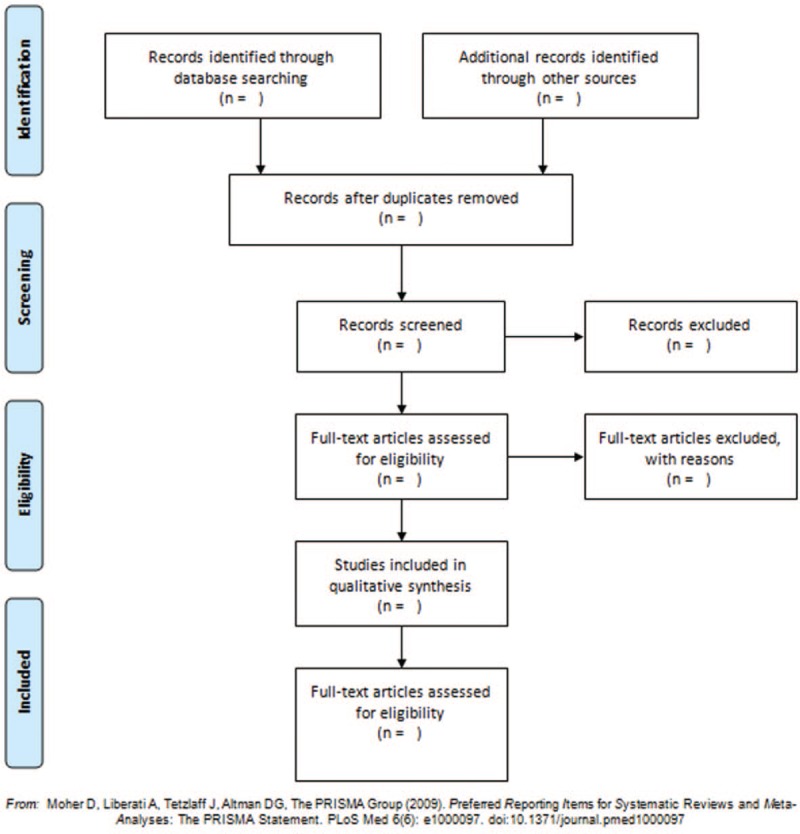
The preferred reporting items for systematic reviews and meta-analyses flow chart.

We search the specified databases to obtain relevant literature, import them into a database created by Endnote X5 and screen out the duplicate documents. Studies will be selected following 2 steps using the program Rayyan.^[13]^ First, studies will be selected by reading titles and abstracts, according to eligibility criteria. Then, the selected studies will be fully read for final step of paper selection and data will be extracted. All steps will be performed by 2 independent reviewers. After the comparison of results, discrepancies will be solved after the analysis of a third reviewer. The screening flow diagrams of this study will be shown in Figure 1.

#### 1.4.2 Data extraction and management

Files will be sent to reviewers for extraction and documentation of data. The following information will be documented after assessing the selected articles:

1. Edition of the publication: Title, journal, author, year, country, type of publication and financing;
2. Study design: type of study, aims, method of data collection and sampling, eligibility criteria, use of instruments/deadline;
3. Study participants: number of study participants and their description, according to: age, sex, indication for warfarin use, duration of warfarin treatment, type and setting of follow-up, presence or absence of comorbidities, types of comorbidities, drugs in chronic use;
4. Evaluation of the instrument used, treatment adherence rate;
5. Limitations: information bias, verification tools, editing tools, tools used for minimum as constraints.

#### 1.4.3 Dealing with missing data

Any missing data or insufficient information will be searched by contacting the paper corresponding author. If those data are not obtained, we will consider only the available data for discussion, emphasizing its impact as a limitation.

#### 1.4.4 Risk of bias of included studies

The risk of bias will be minimized by involving 2 reviewers for blind and independent review of articles. Disagreements will be clarified by discussing with a third reviewer. The following items will be assessed: generation of allocation sequence (selection bias), concealment of allocation sequence (selection bias), blinding (detection and performance bias), blinding of participants and staff for evaluation of results, incomplete data results (attrition bias), reports with selective results (information bias).

#### 1.4.5 Quality appraisal for included studies

The methodological quality of studies will be assessed by using the Newcastle–Ottawa checklist. Score range is 0 to 9 with the following stratification: low quality (≤3 points), medium quality (4–6 points) and high quality (≥7 scores).^[14]^ Any disagreement will be discussed with a third investigator. The results will be summarized on a table and we will evaluate the risk of bias in the selected studies.

#### 1.4.6 Data synthesis and analysis

A quantitative synthesis will be developed if study data is considered sufficiently homogenous. If they are deemed to be heterogeneous, data will be summarized using a narrative (descriptive) synthesis.

## 2 Discussion

Even in the era of direct oral anticoagulants (e.g., dabigatran and rivaroxaban) that present less potential of drug interactions and no requirement of laboratorial monitoring,^[2,3,10]^ warfarin is widely prescribed to prevent thromboembolism. The use of oral anticoagulants is crescent due to the rapid ageing of the population around the globe and the increase of the prevalence of cardiovascular diseases and risk factors for stroke and other thromboembolic complications. In low- and middle-income countries, such as Brazil, warfarin is the main oral anticoagulant available in the public health system. In this context, adherence is crucial to improve effectiveness and safety of warfarin therapy.^[1,15]^

The development of the present systematic will help to summarize and evaluate the validated instruments that have been previously published to assess adherence to warfarin therapy. Besides, this review will substantiate the discussion of relevant topics that should be assessed while providing care to patients taking warfarin. This knowledge will enable a comprehensive approach for healthcare professionals to improve treatment outcomes and the design of future investigations.

## Author contributions

**Conceptualization:** Marcus Fernando da Silva Praxedes, Mayara Sousa Vianna, Maria Auxiliadora Parreiras Martins.

**Funding acquisition:** Maria Auxiliadora Parreiras Martins.

**Investigation:** Marcus Fernando da Silva Praxedes, Mayara Sousa Vianna, Waleska Jaclyn Freitas Nunes de Sousa, Frederico Bartolazzi.

**Methodology:** Marcus Fernando da Silva Praxedes, Mayara Sousa Vianna, Maria Auxiliadora Parreiras Martins.

**Project administration:** Mayara Sousa Vianna.

**Software management for selecting articles:** Marcus Fernando da Silva Praxedes, Mayara Sousa Vianna.

**Supervision:** Maria Auxiliadora Parreiras Martins.

**Writing – original draft:** Marcus Fernando da Silva Praxedes, Mayara Sousa Vianna.

**Writing – review & editing:** Marcus Fernando da Silva Praxedes, Mayara Sousa Vianna, Maria Auxiliadora Parreiras Martins
